# Recombinant PAPP-A resistant insulin-like growth factor binding protein 4 (dBP4) inhibits angiogenesis and metastasis in a murine model of breast cancer

**DOI:** 10.1186/s12885-018-4950-0

**Published:** 2018-10-22

**Authors:** Y. E. Smith, S. Toomey, S. Napoletano, G. Kirwan, C. Schadow, A. J. Chubb, J. H. Mikkelsen, C. Oxvig, J. H. Harmey

**Affiliations:** 1 Angiogenesis and Metastasis Research, Department of Molecular and Cellular Therapeutics, Royal College of Surgeons in Ireland, 123 St. Stephen’s Green, Dublin 2, Ireland; 2 Department of Molecular Biology and Genetics, Aarhus University, Gustav Wieds Vej 10C, 8000 Aarhus C, Denmark

**Keywords:** Angiogenesis, Insulin-like growth factor (IGF), Pregnancy associated plasma protein A/PAPP-A, IGFBP4/dBP4

## Abstract

**Background:**

The Insulin-like growth factor (IGF) pathway plays a role in tumour development and progression. In vivo, IGF1 activity is regulated by the IGF binding proteins (IGFBPs). IGFBP4 inhibits the activity of IGF1 but proteolytic cleavage by pregnancy-associated plasma protein-A (PAPP-A) releases active IGF1. A modified IGFBP4, dBP4, which was resistant to PAPP-A cleavage but retained IGF1 binding capacity, was engineered, expressed in Human Embryonic Kidney (HEK) 293 cells and purified. This study examined the effects of dBP4 on IGF1-induced cell migration, invasion and angiogenesis in vitro. The effect of intra-tumour injections of dBP4 on tumour angiogenesis and metastasis was examined using the 4T1.2luc orthotopic model of breast cancer.

**Methods:**

PAPP-A resistance and IGF binding capacity of dBP4 were characterized by Western blot and surface plasmon resonance, respectively. 4T1.2luc are mouse mammary adenocarcinoma cells transfected with luciferase to allow in vivo imaging. The effect of dBP4 on IGF1-induced Akt activation in 4T1.2luc cells was assessed by Western blot. Cell migration and invasion assays were performed using 4T1.2luc cells. Angiokit™ assays and Matrigel® implants were used to assess the effects of dBP4 on angiogenesis in vitro and in vivo, respectively. An orthotopic breast cancer model – 4T1.2luc cells implanted in the mammary fat pad of BALB/c mice – was used to assess the effect of intra tumour injection of purified dBP4 on tumour angiogenesis and metastasis. Tumour growth and lung metastasis were examined by in vivo imaging and tumour angiogenesis was evaluated by CD31 immunohistochemistry.

**Results:**

Our engineered, PAPP-A resistant IGFBP4 (dBP4) retained IGF1 binding capacity and inhibited IGF1 activation of Akt as well as IGF1-induced migration and invasion by 4T1.2 mammary adenocarcinoma cells. dBP4 inhibited IGF1-induced angiogenesis in vitro and in Matrigel implants in vivo. Direct intra-tumour injection of soluble dBP4 reduced angiogenesis in 4T1.2 luc mammary tumours tumour and reduced lung metastasis.

**Conclusion:**

A PAPP-A resistant IGFBP4, dBP4, inhibits angiogenesis and metastasis in 4T1.2 mammary fat pad tumours. This study highlights the therapeutic potential of dBP4 as an approach to block the tumour-promoting actions of IGF1.

## Background

The insulin-like growth factor (IGF) pathway is involved in proliferation, differentiation, survival, metastasis and drug resistance in various cancers, including breast. The IGF pathway consists of the IGF ligands, IGF1 and 2, their receptors, IGF1R and IGF2R, the insulin receptor (IR), and the IGF binding proteins. Circulating IGF1 and IGF2 are mainly produced by the liver but also by neoplastic tissue [1]. IGF1 binding to the type I IGF receptor (IGF1R), activates downstream signalling pathways, including the phosphatidylinositol-3-kinase (PI3K)/Akt and mitogen-activated protein kinase (MAPK) pathways [2] leading to cellular transformation, proliferation and differentiation [3–5]. Biological activity of IGFs is regulated by the insulin-like binding proteins (IGFBPs) which function as carrier proteins, protecting IGFs from degradation, increasing their half-life and regulating bioavailability. IGFBPs have a higher affinity for IGFs than the IGF1R and the IGFBPs can either inhibit or enhance IGF activity [6–8]. IGFBP4 inhibits IGF1-induced cell proliferation and differentiation in vitro in a number of cell types including bone, muscle and prostate cancer cells [9–14]. IGF is released from IGFBP4 when IGFBP4 is cleaved by the IGFBP4-specific protease, pregnancy-associated plasma protein A (PAPP-A). PAPP-A cleaves IGFBP4 at a single site and its activity is IGF-dependent [15–17]. PAPP-A is expressed by a range of cell types including fibroblasts and osteoblasts [18] and we previously presented evidence that PAPP-A is expressed by host cells within 4T1.2 mammary tumours [19]. There is increasing evidence in the literature that PAPP-A is involved in the growth of a number of different tumour types [20–22]. More recently, a PAPP-A neutralising antibody has shown promise in the treatment of ovarian tumours utilising in vivo models [23].

IGFs are required for normal mammary gland development but are also implicated in the development and maintenance of tumours. We have previously shown that when transfected with a plasmid expressing PAPP-A resistant rat IGFBP4 (dBP4), growth of 4T1.2 cells in the mammary fat pad was inhibited. In addition, there was increased endothelial cell apoptosis within 4T1.2 tumours expressing dBP4 relative to tumours expressing wild type IGFBP4 or transfected with empty vector [19]. Having identified the anti-cancer potential of inhibiting the IGF pathway, we set about to accurately recapitulate the clinical setting through the development of a novel therapeutic for exogenous administration.

Here we show that purified, recombinant murine PAPP-A resistant IGFBP4 (dBP4) retains IGF1 binding capacity and inhibits IGF1 activation of Akt signalling in 4T1.2 cells as well as IGF1-induced cell migration and invasion. dBP4 also inhibited IGF1-induced angiogenesis in vitro and in Matrigel implants in vivo. Direct intra-tumour injection of soluble dBP4 reduced angiogenesis in 4T1.2 luc mammary tumours tumour and reduced lung metastasis. These results demonstrate the therapeutic potential of PAPP-A resistant IGFBP4 as a breast cancer therapeutic.

## Methods

### Cell culture

Murine mammary adenocarcinoma 4T1.2 cells [24] were a gift from Robin Anderson (Peter MacCallum Cancer Centre, Melbourne, Australia) and were cultured in low glucose Dulbecco’s Modified Eagle’s Medium (DMEM) (Biosera, UK), 10% (*v*/v) foetal calf serum (FBS) (Biosera, UK) at 37 °C in 5% (v/v) CO_2_ in air. 4T1.2luc cells were transduced with lentivirus expressing *luc*2 under the control of human ubiquitin C promoter (Caliper, MA, USA). Cells were screened monthly for Mycoplasma contamination.

### dBP4 and wtIGFBP4 protein purification

The PAPP-A cleavage site of mouse wild type IGFBP4 (Accession number NM_010517) was changed from 119-KHMAKVRDRSKMK-133 to 119-AAMAAVADASAMA-133 to generate a cleavage-resistant IGFBP4 variant, which we termed dBP4 (Genscript, UK). The signal sequence was retained and the termination codon removed such that dBP4 was cloned in frame into the XbaI and XhoI sites of pTriEX4neo (Novagen, UK) with a vector-derived 3’HIS tag. wtIGFBP4 and dBP4 were expressed from pTriEx4neo under the control of the CMV promoter with a 3’ HIS-tag. HEK 293 cells which do not express endogenous IGFBP4 were transfected with wtIGFBP4 or dBP4 and selected in G418 (neomycin) to produce a stably transfected cell line designated HEK-wtIGFBP4 or HEK-dBP4. HIS-tagged recombinant protein was purified from serum free conditioned medium using Ni-MAC columns (Novagen, UK). Protein purity was evaluated by Silver staining (Thermo Scientific) following fractionation by SDS-PAGE.

### Migration and invasion assay

10^5^ cells in 200 μl serum free medium (MEMα, (Biosera, UK), 0.05% (*w*/*v*) BSA) were seeded into 8 μm Transwell chambers (Falcon, Becton Dickinson, Ireland) and allowed to adhere for 1 h at 37 °C. Inserts containing the adhered cells were transferred to a second plate and allowed to migrate for 6 h at 37 °C towards 600 μl serum free medium alone, or serum free mediumsupplemented with 5% (*v*/v) FBS, 100 ng/ml (13.16 nM) recombinant human IGF1, or IGF1 (13.16 nM) preincubated with 2.5 μg/ml (73.53 nM) dBP4. Cells were fixed in 10% (v/v) buffered formalin at 4 °C for 16 h. Adhered cells were washed for 5 min in 0.1% (v/v) Triton X-100 followed by 5 min in water. Unmigrated cells were wiped off upper surface of membranes which were then placed on a glass slide in Prolong® gold antifade reagent with DAPI (Invitrogen) and a cover slip. Migrated cells (three fields of view/membrane) were counted under a fluorescent microscope (Olympus, BX51) at 200× magnification using Image J software (NIH, USA). The invasion assay protocol was similar to the migration assay protocol except cells were added to Transwells in Matrigel (1:1 cells in SFM:Matrigel®) (BD Biosciences). Cells were allowed to invade for 16 h at 37 °C and processed as above.

### In vitro angiogenesis assay

TCS cellworks Angiokit™ (TCS cellworks, UK) was used to determine the effect of dBP4 on tubule formation in vitro. Human microvascular endothelial cells were treated with IGF1 (100 ng/ml), dBP4 (2.5 μg/ml) or IGF1 (100 ng/ml) pre-incubated for 1 h with dBP4 (2.5 μg/ml). VEGF (10 ng/ml) and suramin (20 μM) were included as positive and negative controls respectively. Medium was changed every 2–3 days and on day 10 tubules were stained with CD31. Number of tubules and junctions in 3 fields of vie*w*/well (*n* = 3) were scored using the TCS cellworks Angiosystem image analysis software.

### PAPP-A cleavage assay

10 μg purified recombinant dBP4 or recombinant wtIGFBP4 (R&D Systems, UK) was incubated with 10 μg/ml IGF1 (R&D Systems, UK) with or without conditioned medium from cells expressing recombinant PAPP-A for 24 h at 37 °C. Following digestion, IGFBP4 cleavage was analysed by western blot using an anti-IGFBP4 antibody which identifies both the intact protein and the cleavage products (Millipore, Ireland).

### Surface Plasmon resonance

Surface plasmon resonance experiments were carried out on a Biacore T100 instrument (GE Healthcare). Recombinant mouse IGF1, 15 μg/ml in 10 mM sodium acetate pH 4.75, (R&D systems) was coupled to the EDC/NHS activated dextran matrix in flow cell 4 of a series S CM5 sensor chip (GE Healthcare), to a density of 130 response units. Flow cell 3 was left blank to serve as a reference cell. Subsequently, unreacted groups were blocked by a 7 min injection of 1 M ethanolamine, pH 8.0. To collect kinetic binding data, a twofold serial dilution of analyte (mouse wtIGFBP4/dBP4), ranging from 50 nM to 0.4 nM, in 10 mM HEPES pH 7.4, 150 mM NaCl, 1 mM CaCl_2_ and 0.05% Tween-20, was injected over flow cells 3 and 4 at a flow rate of 30 μl/min. The association phase was 120 s, followed by a 240 s dissociation phase. Binding analysis was performed at 37 °C. The surfaces were regenerated by a 60 s injection of 10 mM glycine pH 2.0. Data were collected at a rate of 10 Hz. Recorded signals were subtracted the background signal, as determined by the response obtained from the reference cell. Global fitting of a 1:1 L model was performed, using the Biacore T200 Evaluation Software, version 1.0.

### Western blot analysis

Cells were lysed in RIPA buffer (50 mM TRIS, 150 mM NaCl, 0.5% (*v*/v) NP-40, 1% (v/v) Triton X-100, 0.25% (*w*/*v*) Sodium deoxycholate, 1 mM EDTA pH 7.4, 0.5 mM EGTA pH 7.4) on ice for 30 min. Lysed cells were centrifuged at 10,000 x g for 10 min to remove debris. Protease inhibitor cocktail (1:100 dilution; Sigma Aldrich) was added to cleared lysate. Total protein was quantified using BCA assay (Merck, Germany) or *DC* protein assay (Biorad).

Protein (25 μg) was fractionated by electrophoresis through 4–20% (w/v) SDS-PAGE precast gels (Thermo Scientific, UK) and transferred to nitrocellulose membranes. Membranes were incubated in 5% (w/v) non-fat powdered milk (Marvel, Premier Foods, UK) in TBST (10 mM Tris-HCL, pH 7.4, 100 mM NaCl, 0.1% (*v*/v) Tween-20)) for 1 h. Membranes were washed in TBST then incubated in primary antibody diluted in blocking buffer for 16 h at 4 °C (1:4000 rabbit anti-IGFBP4 (Millipore, Ireland), 1:2000 rabbit anti-Akt (Cell Signalling Technologies, USA), 1:2000 mouse anti-pAkt (ser 473; CST, USA). Membranes were washed 3 times in TBST then incubated in 1:2000 Horse-radish peroxidase (HRP)-conjugated goat anti-rabbit (Dako, Denmark) or goat anti-mouse antibody (Dako, Denmark) 5% (*w*/*v*) non-fat powdered milk TBS-T for 1 h. Membranes were washed 3 times in TBS-T and antibody binding was visualised using ECL reagent (Thermo Scientific, UK).

### Akt activation

4T1.2luc cells were pre-treated with the PI3K inhibitor, wortmannin (500 mM for 1 h) followed by 100 ng/ml IGF1 (13.16 nM) or 100 ng/ml IGF1 (13.16 nM) pre-incubated for 30 min with 2.5 μg/ml dBP4 (73.53 nM) for 0–60 min. Akt and pAkt expression was assessed by western blot.

### In vivo models

Animals were housed in a licensed biomedical research facility (RCSI, St. Stephen’s Green) and had ad libitum access to animal chow. All procedures were reviewed by RCSI Ethics Committee, carried out under animal license guidelines of the Department of Health, Ireland and in accordance with the UK Co-ordinating Committee on Cancer Research (UKCCCR) Guidelines for the Welfare of Animals in Experimental Neoplasia [25].

### Angiogenesis assay

Matrigel® basement membrane matrix (BD Biosciences, Ireland) was placed on ice overnight then mixed with PBS (12.5 μl; vehicle control), VEGF (10 ng/ml; positive control), 100 ng/ml IGF1 (13.16 nM) or 100 ng/ml IGF1 (13.16 nM) pre-incubated with 2.5 μg/ml dBP4 (73.53 nM). 12 week old female BALB/C mice (*n* = 5/group) were anaesthetised under 5% (*v*/v) isoflurane in oxygen. 500 μl of the prepared Matrigel® solution was injected slowly subcutaneously above right hind limb. 7 days later mice were anaesthetised and euthanised by cervical dislocation. Matrigel® implants were excised, fixed in 10% (v/v) buffered formalin overnight then stained with CD31.

### 4T1.2luc model of metastatic breast cancer

5 × 10^4^ 4T1.2luc cells were implanted into the mammary fat pad of 12 week old female BALB/c mice (*n* = 3/group). Tumour growth was monitored by caliper measurements every 2–3 days and the mean tumour diameter (MTD) calculated (square root of the product of length by breadth). When tumours reached a MTD of 8–8.5 mm, mice received intra tumour injections of 50 μg dBP4 or PBS (vehicle control) every 2–3 days. The schedule of injections was chosen after demonstrating that dBP4 remained intact when incubated in mouse plasma for up to 96 h (data not shown). Injections were administered in clockwise rotation, with a single injection per treatment. Mice were paired and treated for 12 (mouse pair 3), 16 (mouse pair 2) or 22 days (mouse pair 3) and the experiment terminated when the tumours in the control animals approached an MTD of 17 mm. Mice were euthanized by cervical dislocation under anaesthesia. 10 min prior to euthanasia, mice received an intraperitoneal injection of 150 mg/Kg luciferin. Primary tumours were imaged prior to euthanasia, and post-mortem lungs were excised and imaged ex vivo using the IVIS (Living Image 3.02 software).

### Immunohistochemistry

Sections (6 μm) were dewaxed with xylene (2 × 4 min), 100% (*v*/v) ethanol (4 × 1 min), 70% (v/v) ethanol (1 min) and dH_2_O (1 min). Antigen retrieval was performed in 0.01 M sodium citrate buffer, pH 6.0 in a steamer for 30 min at 100 °C. Sections were blocked in 3% (v/v) normal goat serum (NGS) (Vector Laboratories, UK) in 0.05% (v/v) PBST for 1 h. Endothelial cells were stained with rabbit anti-CD31 (PECAM) primary antibody diluted 1:100 (Matrigel sections) or 1:200 (4T1.2luc tumours) in blocking buffer overnight at 4 °C. Non-specific rabbit IgG (Dako, Germany) antibody was used as isotype control. Biotin-conjugated goat anti-rabbit secondary antibody (Vector Laboratories, UK) in blocking buffer was incubated for 1 h. Antibody complexes were detected using the ABC reagent kit according to manufacturer’s instructions (Vector Laboratories, UK). Cell nuclei were counterstained with haematoxylin (VWR International, Ireland). Sections were dehydrated in 100% (*v*/v) ethanol (4 × 1 min) xylene (2 × 4 min) then mounted in DPX mounting medium (Sigma Aldrich, Ireland). CD31 positive endothelial cells were quantified in three fields of view (original magnification 200×) for each Matrigel implant (*n* = 5/group) or five fields of view (original magnification 400×) for each tumour (*n* = 3/group) and an average calculated for each group.

## Results

### Purified, recombinant dBP4 binds IGF1 and is resistant to PAPP-A cleavage

**Fig. 1:**
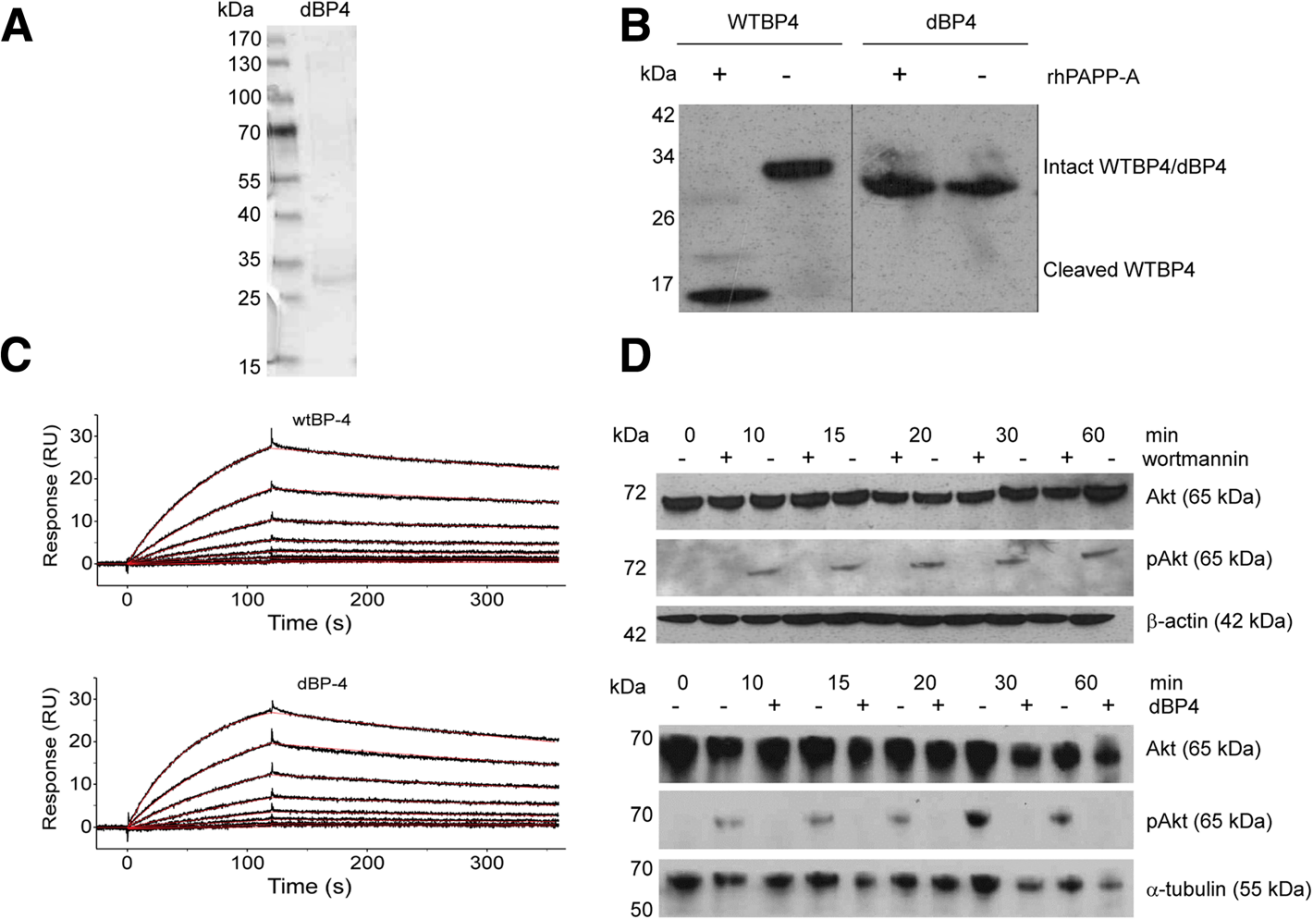
dBP4 binds IGF1, is resistant to PAPP-A cleavage and inhibits IGF-induced Akt phosphorylation. **a** SDS-PAGE analysis of purified dBP4 protein. **b** dBP4 or wtIGFBP4 (WTBP4) were treated with recombinant PAPP-A in the presence of IGF1 for 24 h. Intact IGFBP4 and cleavage fragments were identified by western blot with anti-IGFBP4 antibody. Position of molecular weight (kDa) markers is indicated. Empty lanes on the blot were removed between WTBP4 and dBP4 for clarity. **c** Surface plasmon resonance. Twofold serial dilutions of purified HIS-tagged mouse wtIGFBP4 or dBP4 (50 nM to 0.4 nM) were injected over a surface with immobilized mouse IGF1. Recorded binding curves are shown in black and a global 1:1 L fit is shown in red. **d** 25 μg of cell lysate was fractionated by SDS-PAGE, and total Akt or pAkt identified by western blot. (upper panel) 4T1.2luc cells were pre-treated with the PI3K inhibitor, wortmannin, followed by IGF1 (100 ng/ml). β-actin is shown as loading control. (lower panel) 4T1.2luc cells were treated with IGF1 (100 ng/ml) or IGF1 pre-incubated with 2.5 μg/ml dBP4. α-tubulin is shown as loading control. Blots are representative of three independent experiments

Purified dBP4 was visualized by silver staining following SDS-PAGE fractionation (Fig. 1a). To confirm that mutation of the PAPP-A cleavage site from 119-KHMAKVRDRSKMK-133 to 119-AAMAAVADASAMA-133 rendered IGFBP4 resistant to cleavage by PAPP-A, purified dBP4 was treated with recombinant PAPP-A in the presence of IGF1 and digestion products were identified by Western blotting with anti-IGFBP4 antibody after SDS-PAGE fractionation (Fig. 1b). Although PAPP-A cleaved recombinant wtIGFBP4, with cleavage products visible mainly at 14 kDa and 18 kDa, dBP4 remained intact migrating at approximately 34 kDa. In another unpublished study we found that purified HIS tagged wtIGFBP4 was also cleaved by PAPP-A (data not shown). IGF1 binding affinity of purified wtIGFBP4 and dBP4 was determined by Biacore analysis (Fig. 1c). The fitted constants are: *k*_a_ = 2.110 ± 0.004 × 10^5^ M^− 1^ s^− 1^, *k*_d_ = 8.491 ± 0.038 × 10^− 4^ s^− 1^, resulting in a *K*_D_ = 4.02 × 10^− 9^ M (wtIGFBP4) and *k*_a_ = 3.465 ± 0.005 × 10^5^ M^− 1^ s^− 1^, *k*_d_ = 1.258 ± 0.004 × 10^− 3^ s^− 1^, resulting in a *K*_D_ = 3.63 × 10^− 9^ M (dBP4) showing that IGF1 binding capacity of dBP4 was comparable to wtIGFBP4. These data also demonstrate that the HIS tag and purification protocol did not interfere with IGF binding capacity or susceptibility to PAPP-A cleavage.

### dBP4 inhibits IGF1-induced Akt phosphorylation in 4 T1.2 mammary adenocarcinoma cells

IGF1 treatment for 10 to 60 min resulted in Akt phosphorylation in 4T1.2luc cells (Fig. 1d). Total Akt was unchanged by IGF1 treatment. IGF1 activated Akt in a PI3K-dependent manner as wortmannin abolished IGF1-induced pAkt (upper panel).

4T1.2 cells were treated with recombinant human IGF1 or IGF1 pre-incubated with dBP4. IGF1-induced Akt phosphorylation was abolished by dBP4 demonstrating that dBP4 blocks IGF1 induced activation of Akt in 4T1.2luc cells (lower panel).

### dBP4 inhibits IGF1 induced migration and invasion of 4T1.2luc cells

**Fig. 2:**
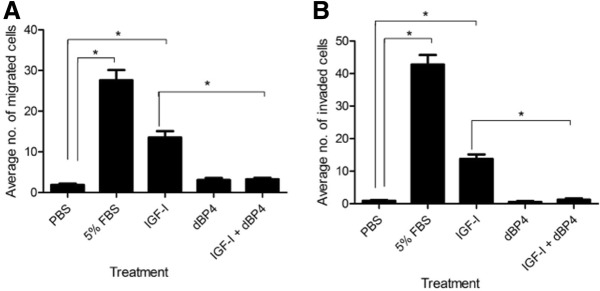
dBP4 inhibits migration and invasion of 4T1.2luc cells. **a** Chemotaxis and **b** invasion of 4T1.2luc cells towards human IGF1 (100 ng/ml), dBP4 (2.5 μg/ml) or IGF1 (100 ng/ml) with dBP4 (2.5 μg/ml). PBS and 5% (*v*/v) FBS were used as negative and positive controls, respectively. Data (*n* = 3 independent experiments) are expressed as mean ± SEM of migrated (**a**) or invaded (**b**) cells from three fields of view (original magnification 200×) (*P < 0.001, one-way ANOVA with Tukey post hoc test)

Migration and invasion are important steps in the formation of metastases in distant organs. Cell migration towards FBS or IGF1 was significantly (*p* < 0.001 and *p* < 0.01, respectively) increased compared to PBS controls. However, when IGF1 was pre-incubated with dBP4, IGF1-induced 4T1.2luc migration was abolished (IGF1 + dBP4 vs. IGF1, *p* < 0.01) (Fig. 2a). dBP4 alone had no significant effect on migration.

FBS or IGF1 significantly increased cell invasion (FBS or IGF1 vs. PBS, *p* < 0.001) (Fig. 2b). When IGF1 was pre-incubated with dBP4 IGF1-induced invasion was abolished (IGF1 + dBP4 vs. IGF1, *p* < 0.001). dBP4 alone had no significant effect on invasion.

### dBP4 inhibits IGF1 induced angiogenesis in vitro

**Fig. 3:**
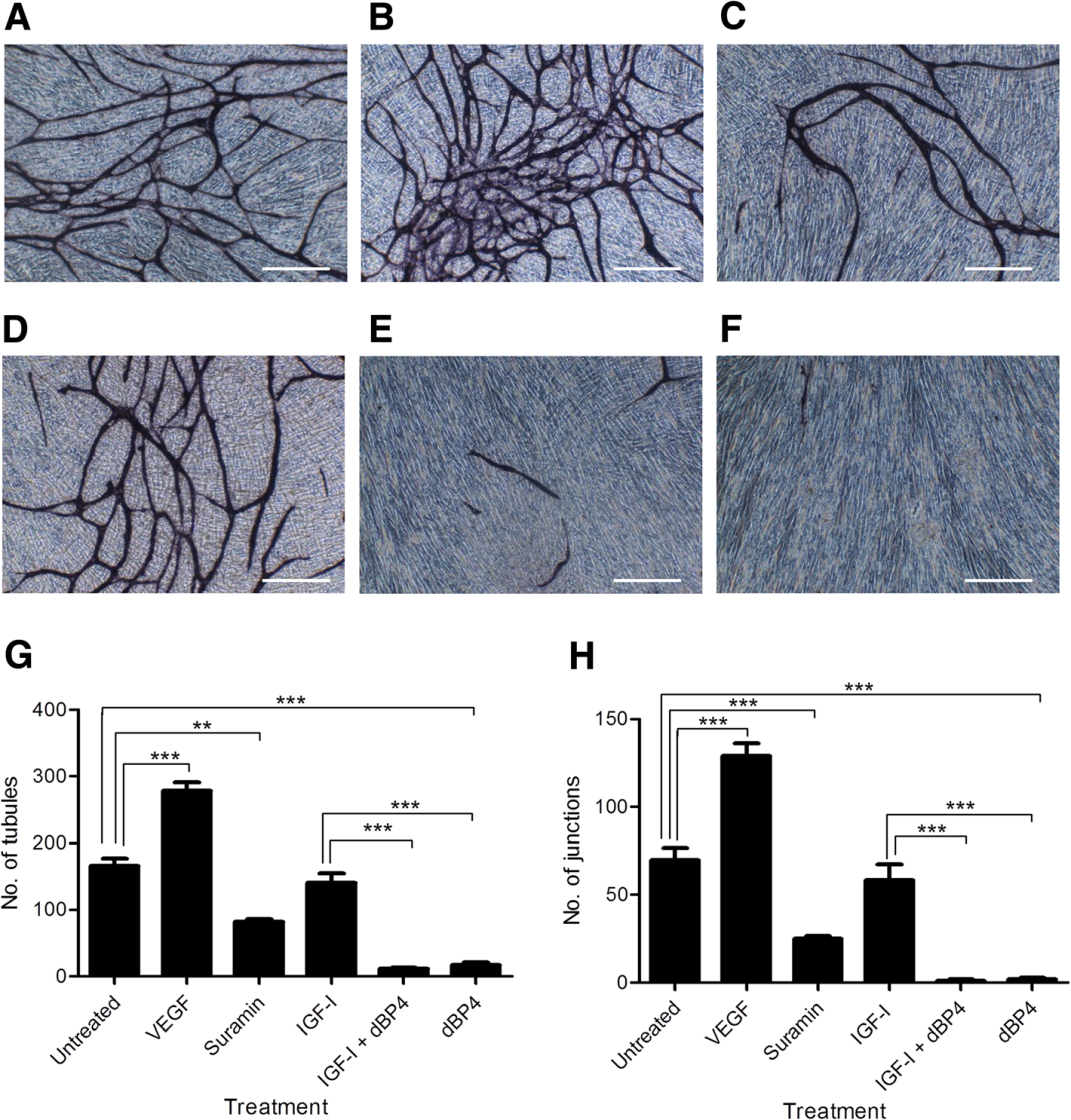
dBP4 inhibits angiogenesis in vitro*.* Representative images of human microvascular endothelial cells stained with CD31. **a** Untreated control, **b** VEGF positive control, **c** suramin negative control, **d** IGF1, **e** IGF1 plus dBP4, **f** dBP4 (scale bar 500 μm). **g** number of tubules and (**h**) number of junctions. Data (n = 3) are expressed as mean ± SEM. (**P < 0.01, *** P < 0.001 one-way ANOVA with Tukey post hoc test)

As dBP4 inhibited 4T1.2luc cell migration and invasion, key steps in metastasis, we also examined the effect of dBP4 on IGF1 induced angiogenesis which is also crucial to metastasis formation. The Angiokit™ is a 24 well plate pre-coated with human endothelial cells co-cultured with other human cells in medium. Following treatment, cells were stained with CD31 to visualise tubule formation. Tubule formation by untreated endothelial cells is shown in Fig. 3a. VEGF, the positive control (Fig. 3b), increased tubule formation relative to the untreated controls (Fig. 3a). Suramin, an angiogenesis inhibitor (Fig. 3c), decreased tubule formation compared to the untreated and VEGF treated controls (Figs. 3a & 2b, respectively). However, IGF1 pre-incubated with dBP4 or dBP4 alone markedly decreased tubule formation compared to the other treatments (Fig. 3e & f, respectively). As dBP4 alone blocked tubule formation it suggests that there is IGF1 present in the assay already from either the growth factor cocktail or produced by the endothelial or other cells present in the assay.

CD31 positive tubules were quantified using the TCS Cellworks Angiosystem image analysis software. Fig. 3g and h show the effect of dBP4 on tubule number and junction number, respectively. Cells treated with VEGF formed significantly more tubules than untreated controls (*p* < 0.001) and suramin decreased tubule number compared to untreated controls (*p* < 0.01). IGF1 treatment did not increase tubule number compared to untreated controls. However, the medium supplied in the Angiokit™ contains a proprietary cocktail of growth factors and it is likely that IGF1 is included in this cocktail possibly accounting for the lack of difference between IGF1 treated and untreated controls. Treatment with IGF1 and dBP4 or dBP4 alone significantly decreased tubule number compared to untreated controls or IGF1-treated cells (*p* < 0.001). These results further suggest the presence of IGF1 in the growth factor cocktail as dBP4 significantly reduced tubule formation in both the presence and absence of exogenous IGF1. When the number of tubule junctions was quantified, a similar effect to that on tubule number was seen. As expected, cells treated with the positive control, VEGF, and the negative control, suramin, had significantly increased or decreased junctions, respectively, compared to untreated controls (*p* < 0.001). IGF1 treated cells did not have a significantly different number of junctions compared to untreated controls. Treatment with IGF1 and dBP4 or dBP4 alone significantly decreased junction numbers compared to IGF1 treated or control cells (*p* < 0.001).

### dBP4 inhibits IGF1 induced angiogenesis in vivo

**Fig. 4:**
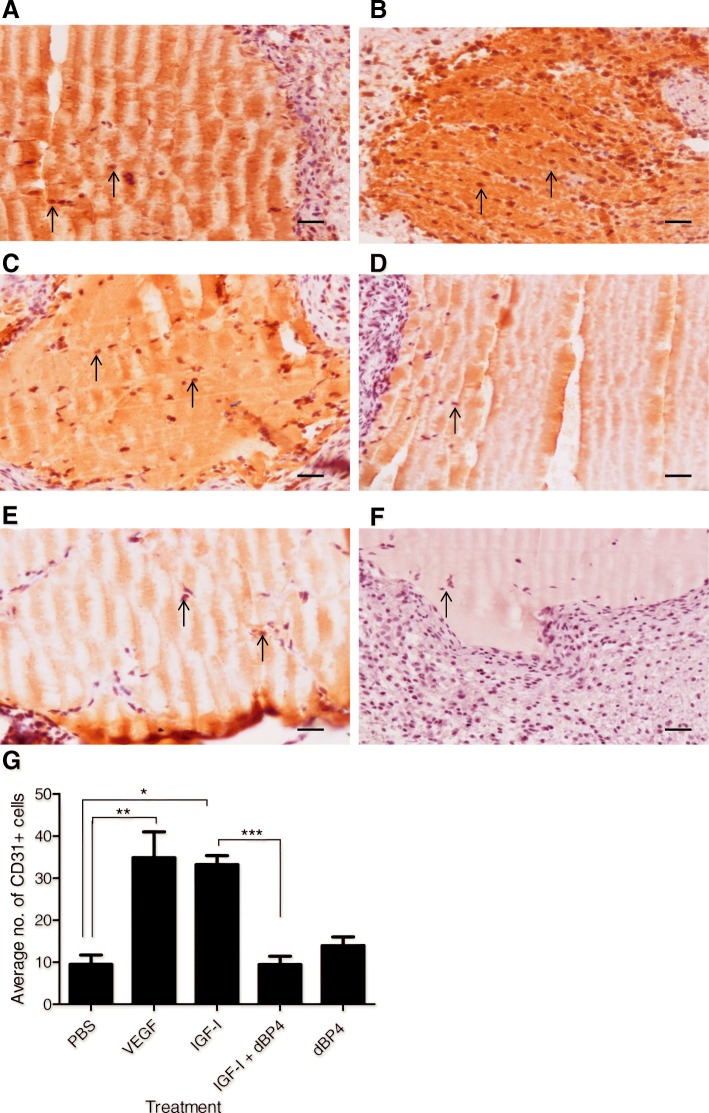
dBP4 inhibits IGF1-induced angiogenesis in vivo*.* 12 week old female BALB/c mice (*n* = 5/group) were injected subcutaneously with Matrigel®. After 7 days implants were excised and stained for CD31 (pointed arrow indicates CD31+ cells). Representative images of **a** PBS negative control, **b** VEGF positive control, **c** IGF1, **d** IGF1 and dBP4, **e** dBP4. **f** shows negative isotype control (scale bars 200 μm). **g** Mean ± SEM CD31+ cells in three fields of view/implant (n = 5). (*P < 0.05), **P < 0.01, ***P < 0.001) one-way ANOVA with Tukey post hoc test)

As dBP4 inhibited tubule formation in vitro, we examined the effects of dBP4 on angiogenesis in vivo using a subcutaneous Matrigel® implant model. Representative images of Matrigel® implants containing PBS (negative control), recombinant human VEGF (10 ng/ml as positive control), recombinant human IGF1 (100 ng/ml), IGF1 (100 ng/ml) pre-incubated with dBP4 (2.5 μg/ml) or dBP4 alone (2.5 μg/ml) are shown in (Fig. 4a-f).

CD31+ cells were counted in 3 fields of view for each Matrigel® implant and (Fig. 4g). Positive control implants containing VEGF had significantly more CD31+ endothelial cells compared to the negative control implants containing PBS (*p* < 0.01). IGF1 implants also had significantly increased endothelial cells compared to PBS controls (*p* < 0.05). Implants containing IGF1 and dBP4 had significantly fewer endothelial cells than IGF1 implants (*p* < 0.001). Implants containing dBP4 alone had comparable numbers of endothelial cells to negative controls (p = n.s.). These results indicate that dBP4 inhibits IGF1-induced angiogenesis in vivo.

### dBP4 inhibits angiogenesis and metastasis of 4T1.2luc tumours

**Fig. 5:**
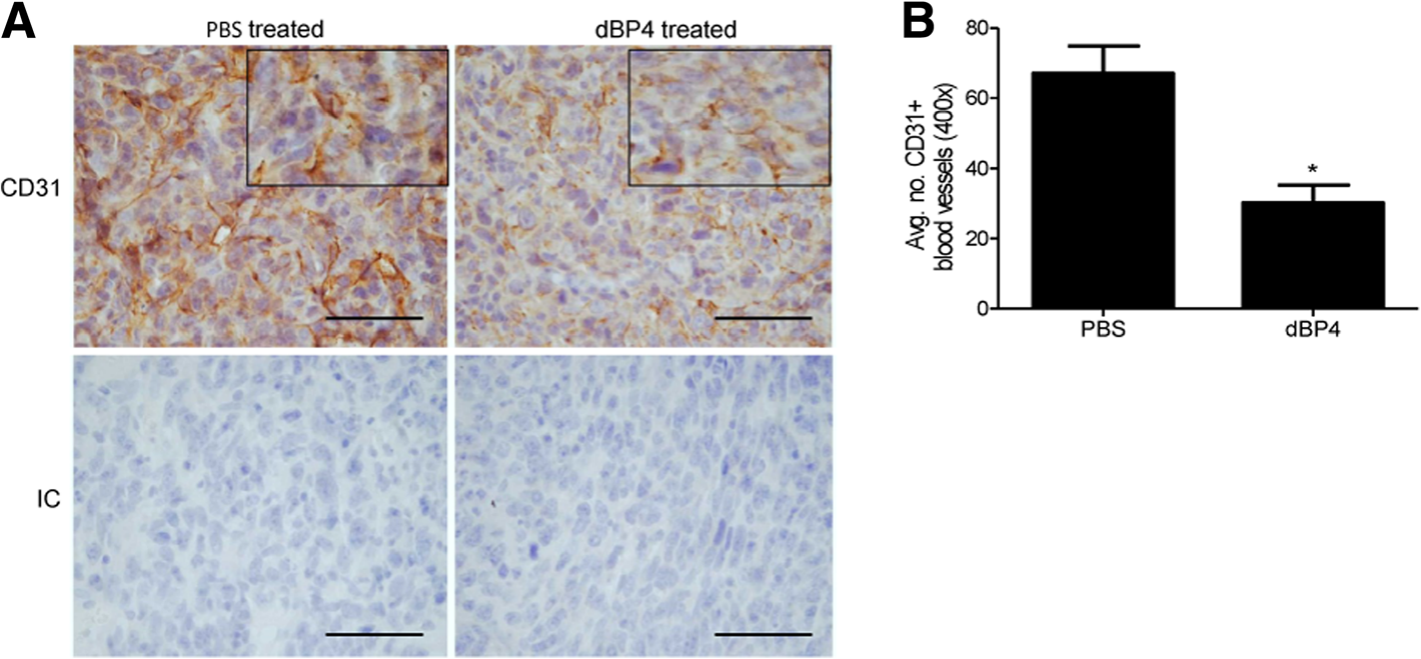
dBP4 inhibits angiogenesis in 4T1.2luc tumours. 4T1.2luc cells (5 × 10^4^) were implanted into the mammary fat pad of female BALB/c mice (n = 3/group). Once tumours reached a MTD of 8–8.5 mm, mice received an intra-tumour injection of 50 μg dBP4 or PBS every 2–3 days. Once tumours reached a MTD of 17 mm, mice were sacrificed. Primary tumours were excised and blood vessels visualized by staining for CD31 **a** shows representative images of CD31+ stained tumours from PBS or dBP4 treated mice. IC: Negative isotype control. (scale bar 50 μm). Enlarged area shows vessel morphology. **b** Mean ± SEM CD31+ vessels in 5 fields of view/tumour (n = 3/group). (*P < 0.05, unpaired Students t-test)

The preceding data and our previous study using 4T1.2 cells transfected with dBP4 expression plasmid [19] suggested that administration of purified dBP4 protein would inhibit tumour angiogenesis. As a precursor to future studies to examine the effects of systemic administration, we conducted a pilot study using direct intra-tumour injection of purified, dBP4. 4T1.2luc cells were implanted into the mammary fat pad of female BALB/c mice (*n* = 3/group). When tumours reached a mean tumour diameter (MTD) of 8–8.5 mm, mice received intra tumour injections of 50 μg purified, recombinant dBP4 or PBS (vehicle control) every 2–3 days. Mice were culled once a maximum MTD of 17 mm was reached from either group. Following treatment, control and dBP4-treated tumours were stained for CD31 and the blood vessels were quantified. Representative images of CD31 stained control and dBP4-treated tumours are shown in Fig. 5a. dBP4 treated tumours had significantly fewer blood vessels than PBS treated tumours (Fig. 5b). The vessels in dBP4-treated tumours appeared broken and incomplete compared to PBS treated tumours, as shown in the enlarged area in the upper panels of Fig. 5a. These results indicate that dBP4 treatment decreased angiogenesis within 4T1.2luc2 mammary tumours.

**Fig. 6:**
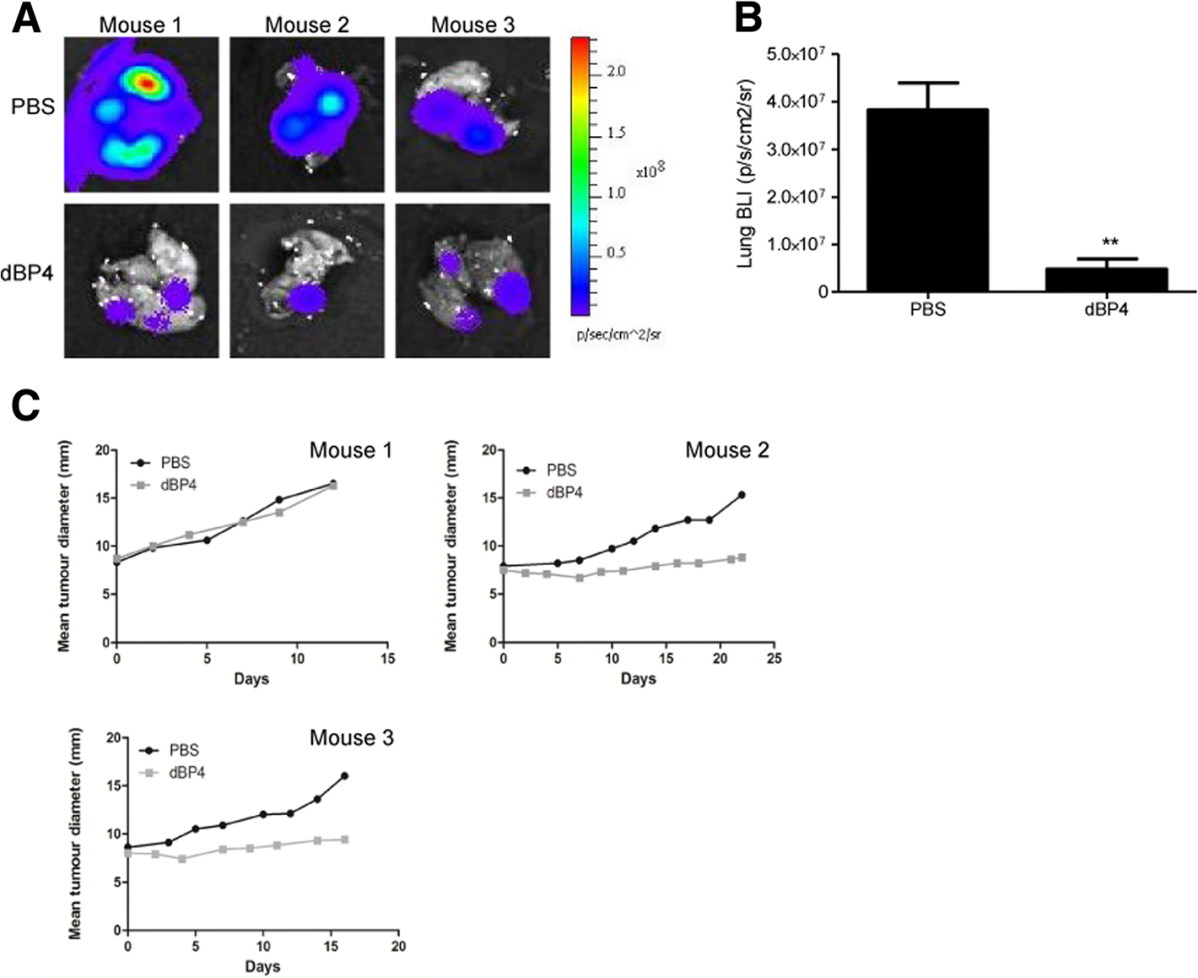
**a** Bioluminescence (BLI) images of lungs from dBP4 or PBS treated mice (n = 3). **b** Metastatic lung burden following treatment expressed as BLI. Data are expressed as mean ± SEM (*P < 0.05, **P < 0.01, unpaired Students t test). **c** Primary tumour growth curves of individual mice

dBP4 treatment significantly decreased metastatic burden compared to PBS treated controls (*p* < 0.01), based on BLI imaging of lungs ex vivo following treatment (Fig. 6a and b). Treatment with dBP4 inhibited primary tumour growth in two of three mice compared to PBS treated mice (Fig. 6c).

## Discussion

The activity of IGF is regulated by the IGF binding proteins which can either inhibit or potentiate IGF activity. When bound to IGFBP4, IGF is inactive and active IGF is released when IGFBP4 is cleaved by PAPP-A [26]. We mutated the PAPP-A cleavage site of mouse IGFBP4 and cloned the construct, dBP4, into pTriExneo expression vector with a 3’ HIS tag to facilitate purification. Treatment of wtIGFBP4 with PAPP-A resulted in proteolytic cleavage, whereas dBP4 was resistant to PAPP-A cleavage. Surface Plasmon Resonance analysis demonstrated that IGF binding capacity of dBP4 was comparable to previously published values for wtIGFBP4 [27].

We previously demonstrated that IGF1 increased proliferation (by 50%) of 4T1.2 cells and to a much greater extent (approx. 400%) proliferation of microvascular endothelial cells [19]. Here we have shown that IGF1 activates Akt in a PI3K dependent manner in 4T1.2luc cells as the PI3K inhibitor, wortmannin, abolished IGF1-induced pAkt. Treatment with dBP4 blocked Akt activation by IGF1.

Angiogenesis is essential for the survival and metastasis of tumours [28]. We previously demonstrated that wild type IGFBP4 inhibited IGF1-induced proliferation of microvascular endothelial cells in vitro and that IGF1 increased VEGF production by 4T1.2 mammary adenocarcinoma cells [19], suggesting that dBP4 would have anti-angiogenic effects. In a multicellular in vitro angiogenesis assay, dBP4 either alone or in the presence of exogenous IGF1 inhibited tubule formation. Exogenous IGF1 alone had no effect and dBP4 alone inhibited tubule formation, suggesting the presence of endogenous IGF1 in the assay from either the growth factor cocktail or produced by cells present in the assay. However, both wild type IGBP4 and a C-terminal fragment of IGFBP4 inhibited VEGF-induced angiogenesis in an in vitro Matrigel assay [29]. Although the mechanisms of IGF-independent activity of IGFBP4 are not fully understood, Moreno et al. implicated cathepsin inhibition by the C terminal region of IGFBP4 [29]. In contrast,wild type IGFBP4 inhibited IGF1 or FGF-2 induced, but not VEGF-induced angiogenesis in an in vivo CAM (chick chorioallantoic membrane) angiogenesis model [30]. It is therefore possible that the observed reduction in tubule formation by dBP4 in the absence of exogenous IGF1 is due to presence of endogenous IGF1, IGF1-independent effects or VEGF-dependent effects. A recent review has highlighted the importance of context (when, where, how, how much and what else) to explain apparently conflicting results in the IGF1 signalling literature [31].

We have shown here that dBP4 inhibited IGF1-mediated angiogenesis in Matrigel® subcutaneous implants. In contrast to the in vitro angiogenesis data where dBP4 reduced angiogenesis in the absence of exogenous IGF1 most likely due to an inhibitory effect on the cocktail of growth factors present, angiogenesis in Matrigel implants with dBP4 in the absence of exogenous growth factors was similar to Matrigel controls.

We examined the effects of purified dBP4 on tumour angiogenesis and metastasis using direct intra-tumour injections in the 4T1.2luc model of metastatic breast cancer. For this preliminary study we chose intra-tumour injection of purified dBP4 every 2–3 days to ensure dBP4 reached the tumour and avoided confounding variables of systemic delivery systems. A recent review has highlighted differences between locally and systemically delivered IGF1 in other disease models [31] so our future studies will include comparisons between intra-tumour (local) and systemic delivery. We had established that dBP4 was stable in mouse serum from healthy and 4T1.2 tumour bearing mice at 37 °C for over 4 days (data not shown). 4T1.2luc mammary adenocarcinoma tumours were established in wild type BALB/c mice before being treated with purified recombinant dBP4 allowing the effect of exogenously administered dBP4 to be evaluated in a clinically relevant model.

Tumours treated with dBP4 had significantly fewer blood vessels compared to PBS treated tumours. Blood vessels appeared broken and incomplete and large areas of necrosis were visible in dBP4 treated tumours. We previously observed that vessels in 4T1.2 tumours constitutively expressing rat dBP4 were of poor quality with occluded lumens [19]. It has been established that anti-angiogenic therapies result in tumour necrosis which eventually causes the centre of a tumour to become hollow (cavitation), a process which may explain why there was no significant reduction in primary tumour volume, despitedecreased vascularisation and lung metastasis [32]. Although 4T1.2 cells do not produce PAPP-A, host cells within 4T1.2 tumours do produce PAPP-A [19] and is the likely reason why wtIGFBP4 had no effect on 4T1.2 tumour growth or angiogenesis in that study as host-cell derived PAPP-A will cleave the wtIGFBP4 rendering it unable to sequester IGF. The differing reports on the effects of wtIGFBP4 and dBP4 on tumour growth and angiogenesis most likely depend on whether PAPP-A is expressed by the tumour cells or tumour microenvironment [13, 19, 33].

In addition to inhibiting angiogenesis within primary mammary tumours dBP4 treatment reduced lung metastatic burden with no obvious toxicity. The IGF pathway has previously been implicated in breast cancer metastasis. IGF1 increases cell migration and invasion in vitro in a range of cancer cells, including human colorectal carcinoma and human multiple myeloma cells [34, 35]. IGF1 increased motility of metastatic MDA-MB-231BO breast cancer cells through activation of the IGF1R [36] and a dominant negative mutant of IGF1R decreased adhesion, invasion and metastasis of MDA-MB-435 cells [37]. In vitro treatment of 4T1.2luc cells with dBP4 inhibited IGF1 induced migration and invasion. The reduction in lung metastases in dBP4 mice may be secondary toangiogenesis inhibition in the primary tumour and/or inhibition of tumour cell migration and invasion.

Several strategies targeting IGF1 directly have proved successful in in vivo studies. The involvement of the IGF pathway in cancer has led to the investigation of other components of the pathway such as IGFBP4 and PAPP-A. The results of those studies support the findings we present here. Infusion of a rat protease resistant IGFBP4 into the perilesion environment in a porcine model of neointimal hyperplasia inhibited IGF1 and neointimal expansion [38]. In a transgenic mouse model where both human protease resistant IGFBP4 and PAPP-A were overexpressed in osteoblasts, the protease resistant form of IGFBP4 inhibited PAPP-A mediated bone formation through sequestration of IGF [39]. Overexpression of PAPP-A in SKOV3 cells increased anchorage-independent growth and invasion in vitro and promoted tumour growth and vascularisation in vivo compared to a mutated version with decreased protease activity [21]. PAPP-A inhibition by a monoclonal antibody inhibited IGF1-induced Akt activation and intraperitoneal administration inhibited growth of A549 xenografts [40]. A recent study identified high levels of PAPP-A in the ascites of ovarian cancer patients and more cleaved than intact IGFBP4, indicating that PAPP-A increased IGF activity and therefore stimulated tumour growth via IGF1-R stimulation [41]. Our approach was to use dBP4 to sequester IGF1 rather than blocking PAPP-A itself.

The data presented here demonstrate that a purified, recombinant PAPP-A-resistant IGFBP4, dBP4, inhibits IGF1-induced angiogenesis in vitro and in vivo. Direct injection of purified dBP4 into 4T1.2 tumours inhibited both angiogenesis and metastasis of mammary 4T1.2luc tumours. Future studies will optimise large scale production of dBP4 and evaluate systemic delivery systems such as osmotic pumps to thoroughly assess the clinical potential of dBP4 as a cancer therapeutic. dBP4 is small enough to cross the endothelial cell barrier [42] and due to its resistance to PAPP-A cleavage we anticipate a long half-life in the circulation. As a way of blocking the IGF pathway, dBP4 via sequestering IGF-I may have advantages over monoclonal antibody approaches blocking IGF, IGF1-R or PAPP-A directly.

## Conclusion

These studies demonstrated that a PAPP-A resistant IGFBP4, dBP4, inhibits angiogenesis and metastasis in 4T1.2 mammary fat pad tumours. The data highlight the therapeutic potential of dBP4 as an approach to blocking the tumour-promoting actions of IGF1.

## Abbreviations

dBP4: PAPP-A resistant IGFBP4
IGF: Insulin-like growth factor
IGF1-R: IGF1 receptor
IGFBP4: IGF binding protein 4
PAPP-A: Pregnancy associated plasma protein A

## References

[1] Pollak M (2008). Insulin and insulin-like growth factor signalling in neoplasia. Nat Rev Cancer.

[2] Leroith D, Werner H, Beitner-Johnson D, Roberts CT (1995). Molecular and cellular aspects of the insulin-like growth factor I receptor. Endocr Rev.

[3] Petley T, Graff K, Jiang W, Yang H, Florini J (1999). Variation among cell types in the signaling pathways by which IGF1 stimulates specific cellular responses. Horm Metab Res.

[4] Hermanto U, Zong CS, Wang LH (2000). Inhibition of mitogen-activated protein kinase kinase selectively inhibits cell proliferation in human breast cancer cells displaying enhanced insulin-like growth factor I-mediated mitogen-activated protein kinase activation. Cell Growth Differ.

[5] Grey A, Chen Q, Xu X, Callon K, Cornish J (2003). Parallel phosphatidylinositol-3 kinase and p42/44 mitogen-activated protein kinase signaling pathways subserve the mitogenic and antiapoptotic actions of insulin-like growth factor I in osteoblastic cells. Endocrinology.

[6] Clemmons DR (1997). Insulin-like growth factor binding proteins and their role in controlling IGF actions. Cytokine Growth Factor Rev.

[7] Kelley KM, Oh Y, Gargosky SE, Gucev Z, Matsumoto T, Hwa V, Ng L, Simpson DM, Rosenfeld RG (1996). Insulin-like growth factor-binding proteins (IGFBPs) and their regulatory dynamics. Int J Biochem Cell Biol.

[8] Khandwala HM, Mccutcheon IE, Flyvbjerg A, Friend KE (2000). The effects of insulin-like growth factors on tumorigenesis and neoplastic growth. Endocr Rev.

[9] Schiltz PM, Mohan S, Baylink DJ (1993). Insulin-like growth factor binding protein-4 inhibits both basal and IGF-mediated chick pelvic cartilage growth in vitro. J Bone Miner Res.

[10] Mohan S, Nakao Y, Honda Y, Landale E, Leser U, Dony C, Lang K, Baylink DJ (1995). Studies on the mechanisms by which insulin-like growth factor (IGF) binding protein-4 (IGFBP-4) and IGFBP-5 modulate IGF actions in bone cells. J Biol Chem.

[11] Mohan S, Baylink DJ (2002). IGF-binding proteins are multifunctional and act via IGF-dependent and -independent mechanisms. J Endocrinol.

[12] Damon SE, Haugk KL, Birnbaum RS Quinn LS (1998). Retrovirally mediated overexpression of insulin-like growth factor binding protein 4: evidence that insulin-like growth factor is required for skeletal muscle differentiation. J Cell Physiol.

[13] Damon SE, Maddison L, Ware JL, Plymate SR (1998). Overexpression of an inhibitory insulin-like growth factor binding protein (IGFBP), IGFBP-4, delays onset of prostate tumor formation. Endocrinology.

[14] Gustafsson T, Andersson P, Arnqvist HJ (1999). Different inhibitory actions of IGFBP-1, −2 and −4 on IGF1 effects in vascular smooth muscle cells. J Endocrinol.

[15] Byun D, Mohan S, Kim C, Suh K, Yoo M, Lee H, Baylink DJ, Qin X (2000). Studies on human pregnancy-induced insulin-like growth factor (IGF)-binding protein-4 proteases in serum: determination of IGF1I dependency and localization of cleavage site. J Clin Endocrinol Metab.

[16] Laursen LS, Overgaard MT, Soe R, Boldt HB, Sottrup-Jensen L, Giudice LC, Conover CA, Oxvig C (2001). Pregnancy-associated plasma protein-a (PAPP-A) cleaves insulin-like growth factor binding protein (IGFBP)-5 independent of IGF: implications for the mechanism of IGFBP-4 proteolysis by PAPP-A. FEBS Lett.

[17] Laursen LS, Overgaard MT, Nielsen CG, Boldt HB, Hopmann KH, Conover CA, Sottrup-Jensen L, Giudice LC, Oxvig C (2002). Substrate specificity of the metalloproteinase pregnancy-associated plasma protein-a (PAPP-A) assessed by mutagenesis and analysis of synthetic peptides: substrate residues distant from the scissile bond are critical for proteolysis. Biochem J.

[18] Durham SK, Riggs BL, Harris SA, Conover CA (1995). Alterations in insulin-like growth factor (IGF)-dependent IGF-binding protein-4 proteolysis in transformed osteoblastic cells. Endocrinology.

[19] Ryan AJ, Napoletano S, Fitzpatrick PA, Currid CA, O'Sullivan NC, Harmey JH (2009). Expression of a protease-resistant insulin-like growth factor-binding protein-4 inhibits tumour growth in a murine model of breast cancer. Br J Cancer.

[20] Bulut I, Coskun A, Ciftci A, Cetinkaya E, Altiay G, Caglar T, Gulcan E (2009). Relationship between pregnancy-associated plasma protein-a and lung cancer. Am J Med Sci.

[21] Boldt HB, Conover CA (2011). Overexpression of pregnancy-associated plasma protein-a in ovarian Cancer cells promotes tumor growth in vivo. Endocrinology.

[22] Huang J, Tabata S, Kakiuchi S, The Van T, Goto H, Hanibuchi M, Nishioka Y (2013). Identification of pregnancy-associated plasma protein a as a migration-promoting gene in malignant pleural mesothelioma cells: a potential therapeutic target. Oncotarget.

[23] Becker MA, Haluska P, Bale LK, Oxvig C, Conover CA (2015). A novel neutralizing antibody targeting pregnancy-associated plasma protein-a inhibits ovarian Cancer growth and ascites accumulation in patient mouse Tumorgrafts. Mol Cancer Ther.

[24] Lelekakis M, Moseley JM, Martin TJ, Hards D, Williams E, Ho P, Lowen D, Javni J, Miller FR, Slavin J, Anderson RL (1999). A novel orthotopic model of breast cancer metastasis to bone. Clin Exp Metastasis.

[25] UKCCCR. United Kingdom (1998). Co-ordinating committee on Cancer research (UKCCCR) guidelines for the welfare of animals in experimental Neoplasia (second edition). Br J Cancer.

[26] Conover CA, Bale LK, Overgaard MT, Johnstone EW, Laursen UH, Füchtbauer EM, Oxvig C, van Deursen J (2004). Metalloproteinase pregnancy-associated plasma protein a is a critical growth regulatory factor during fetal development. Development.

[27] Laursen LS, Kjaer-Sorensen K, Andersen MH, Oxvig C (2007). Regulation of insulin-like growth factor (IGF) bioactivity by sequential proteolytic cleavage of IGF binding protein-4 and -5. Mol Endocrinol.

[28] Folkman J, Shing Y (1992). Angiogenesis. J Biol Chem.

[29] MJ M, Ball M, Rukhlova M, Slinn J, L'abbe D, Iqbal U, Monette R, Hagedorn M, O'Connor-McCourt MD, Durocher Y, Stanimirovic DB (2013). IGFBP-4 anti-angiogenic and anti-tumorigenic effects are associated with anti-cathepsin B activity. Neoplasia.

[30] Contois LW, Nugent DP, Caron JM, Cretu A, Tweedie E, Akalu A, Liebes L, Friesel R, Rosen C, Vary C, Brooks PC (2012). Insulin-like growth factor binding protein-4 differentially inhibits growth factor-induced angiogenesis. J Biol Chem.

[31] Conover CA (2016). Discrepancies in insulin-like growth factor signaling? No, not really. Growth Hormon IGF Res.

[32] Crabb SJ, Patsios D, Sauerbrei E, Ellis PM, Arnold A, Goss G, Leighl NB, Shepherd FA, Powers J, Seymour L, Laurie SA (2009). Tumor cavitation: impact on objective response evaluation in trials of angiogenesis inhibitors in non-small-cell lung cancer. J Clin Oncol.

[33] Englemann JC, Amann T, Ott-Rötzer B, Nützel M, Reinders Y, Reinders J, Thasler WE, Kristl T, Teufel A, Huber CG, Oefner PJ, Spang R, Hellerbrand C (2015). Causal modeling of Cancer-stromal communication identifies PAPPA as a novel Stroma-secreted factor activating NFκB signaling in hepatocellular carcinoma. PLoS Comput Biol.

[34] Bauer TW, Fan F, Liu W, Johnson M, Parikh NU, Parry GC, Callahan J, Mazar AP, Gallick GE, Ellis LM (2005). Insulin like growth factor-I-mediated migration and invasion of human colon carcinoma cells requires activation of c-met and urokinase plasminogen activator receptor. Ann Surg.

[35] Qiang YW, Yao L, Tosato G, Rudikoff S (2004). Insulin-like growth factor I induces migration and invasion of human multiple myeloma cells. Blood.

[36] Jackson JG, Zhang X, Yoneda T, Yee D (2001). Regulation of breast cancer cell motility by insulin receptor substrate-2 (IRS-2) in metastatic variants of human breast cancer cell lines. Oncogene.

[37] Dunn SE, Ehrlich M, Sharp NJ, Reiss K, Solomon G, Hawkins R, Baserga R, Barrett JC (1998). A dominant negative mutant of the insulin-like growth factor-I receptor inhibits the adhesion, invasion, and metastasis of breast cancer. Cancer Res.

[38] Nichols TC, Busby WHJ, Merricks E, Sipos J, Rowland M, Sitko K, Clemmons DR (2007). Protease-resistant insulin-like growth factor (IGF)-binding protein-4 inhibits IGF1 actions and neointimal expansion in a porcine model of neointimal hyperplasia. Endocrinology.

[39] Phang D, Rehage M, Bonafede B, Hou D, Xing W, Mohan S, Wergedal JE, Qin X (2010). Inactivation of insulin-like-growth factors diminished the anabolic effects of pregnancy-associated plasma protein-a (PAPP-A) on bone in mice. Growth Hormon IGF Res.

[40] Mikkelsen JH, Resch ZT, Kalra B, Savjani G, Kumar A, Conover CA, Oxvig C (2014). Indirect targeting of IGF receptor signalling in vivo by substrate-selective inhibition of PAPP-A proteolytic activity. Oncotarget.

[41] Thomsen J, Hjortebjerg R, Espelund U, Ørtoft G, Vestergaard P, Magnusson NE, Conover CA, Tramm T, Hager H, Høgdall C, Høgdall E, Oxvig C, Frystyk J (2015). PAPP-A proteolytic activity enhances IGF bioactivity in ascites from women with ovarian carcinoma. Oncotarget.

[42] Boes M, Booth BA, Sandra A, Dake BL, Bergold A, Bar RS (1992). Insulin-like growth factor binding protein (IGFBP)4 accounts for the connective tissue distribution of endothelial cell IGFBPs perfused through the isolated heart. Endocrinology.

